# Identification of Nonvolatile Migrates from Food Contact Materials Using Ion Mobility–High-Resolution Mass Spectrometry and in Silico Prediction Tools

**DOI:** 10.1021/acs.jafc.2c03615

**Published:** 2022-07-20

**Authors:** Xue-Chao Song, Elena Canellas, Nicola Dreolin, Jeff Goshawk, Cristina Nerin

**Affiliations:** † Department of Analytical Chemistry, Aragon Institute of Engineering Research I3A, CPS-University of Zaragoza, Maria de Luna 3, 50018 Zaragoza, Spain; ‡ Waters Corporation, Altrincham Road, SK9 4AX Wilmslow, United Kingdom

**Keywords:** ion mobility, collision cross section, machine learning, in silico tools, retention time prediction, food safety, food contact materials, migration, polyamide

## Abstract

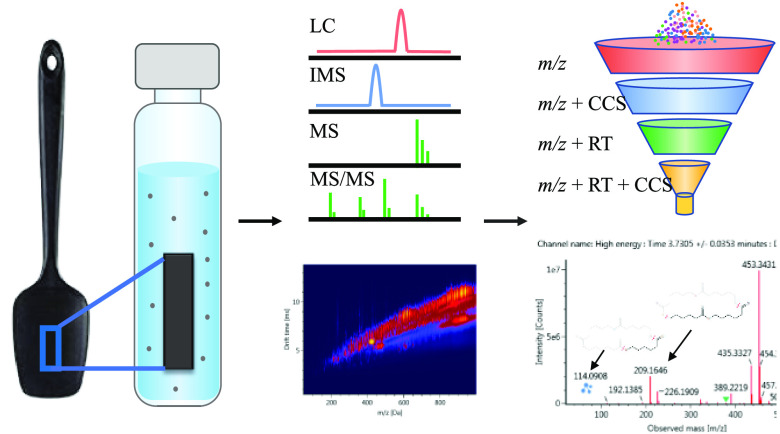

The identification of migrates from food contact materials (FCMs) is challenging due to the complex matrices and limited availability of commercial standards. The use of machine-learning-based prediction tools can help in the identification of such compounds. This study presents a workflow to identify nonvolatile migrates from FCMs based on liquid chromatography–ion mobility–high-resolution mass spectrometry together with in silico retention time (RT) and collision cross section (CCS) prediction tools. The applicability of this workflow was evaluated by screening the chemicals that migrated from polyamide (PA) spatulas. The number of candidate compounds was reduced by approximately 75% and 29% on applying RT and CCS prediction filters, respectively. A total of 95 compounds were identified in the PA spatulas of which 54 compounds were confirmed using reference standards. The development of a database containing predicted RT and CCS values of compounds related to FCMs can aid in the identification of chemicals in FCMs.

## Introduction

Foods and beverages can come into contact with a range of materials during production, transport, storage, and serving. Such food contact materials (FCMs) contain chemicals, termed food contact chemicals (FCCs), that can migrate into the food and pose health concerns for consumers.^1^ It has been estimated that the contamination of food by organic chemicals from FCMs may be 100 times higher than that from pesticides and other environmental pollutants.^2^ Although FCMs include paper, board, glass, metal, biomaterials, and multilayers, it is plastic polymers that predominantly come into contact with food.^3^ In the production of plastics, additives are used to enhance the performance of the final product. The most commonly used additives include plasticizers, antioxidants, stabilizers, flame retardants, slip agents, and lubricants. These additives are not always chemically bound to the polymers and, as such, can migrate into food.^4^

FCMs can also contain nonintentionally added substances (NIAS) which are chemical contaminants, generally formed by the degradation products of additives or the polymer itself, side products, or reaction products. The degradation products of additives have been widely studied by different research groups.^5−8^ Oligomers are commonly present in polymers originating from incomplete polymerization or the degradation of polymer chains.^9^ Studies have been published on the oligomers of polylactic acid (PLA),^10^ polyethylene terephthalate (PET),^11,12^ and polyurethane (PU) adhesive.^13^

Full identification of FCCs originating from polymeric FCMs is challenging due to the complexity of plastic matrices, the unknown, or unexpected, constituents of the plastic, as well as the limited availability of reference standards. High-resolution mass spectrometry (HRMS), including time-of-flight (TOF), has been used for the identification of nonvolatile FCCs,^14−18^ where the elemental composition of detected compounds can be determined from the precursor ion and isotopic distribution. However, upon searching for compounds with a given molecular formula in public scientific databases, such as ChemSpider or PubChem, an extensive number of potential structures can be found. In such cases, the experience of analysts and their skill in MS spectral interpretation are essential for eliminating unlikely entries and reducing false positives.

In recent years, the hyphenation of ultra-high-performance liquid chromatography with ion mobility spectroscopy (IMS) and quadrupole time-of-flight mass spectrometry (UPLC-IMS-QTOF) has been widely used for the targeted and untargeted screening of complex samples.^19−23^ The collision cross section (CCS), derived from IMS, is a stable parameter related to the size, shape, and charge of a gas-phase ion.^24^ Excellent interday precision of CCS measurements was observed in the studies by Regueiro et al.^25^ and Song et al.,^26^ with relative standard deviation (RSD) values below 1% for pesticides and FCCs, respectively. Righetti et al.^27^ have shown that the CCS values of mycotoxins measured from two IMS platforms located in two different laboratories varied only within 1.5%. The consistent reproducibility of CCS measurements makes them an ideal complement to retention time (RT), accurate mass, isotopic pattern, and fragment ions for the identification of compounds.^22^

A limitation in the identification of FCCs originating from FCMs is that many of the suspect compounds are not commercially available; thus, tentative and probable identification cannot be confirmed by comparing the RT, MS spectra, and CCS values of unknowns to those of reference standards. To mitigate this situation, some research groups have developed RT and CCS prediction models using machine learning approaches. Bonini and co-workers developed an R package, *Retip*, that predicts RT values for metabolomics studies, and they found that 68% of candidate structures were filtered out with the addition of RT match.^28^ CCS prediction tools, such as AllCCS,^29^ CCSondemand,^30^ CCSbase,^31^ and DeepCCS, have been developed by a number of research groups.^32^ Zhou et al.^29^ have shown that the number of candidates are reduced by approximately 75% with the addition of a CCS match. In addition to the significant reduction of candidates facilitated by RT and CCS agreement, the comparison between experimental and predicted RT and CCS values for which both RT and CCS values are within tolerance can increase the confidence of the identification, especially in absence of reference standards.

Two databases related to FCMs are the Chemicals associated with Plastic Packaging Database (CPPdb)^33^ and the Food Contact Chemicals Database (FCCdb) (https://www.foodpackagingforum.org/fccdb);^34^ both were compiled by Groh and co-workers. CPPdb contains 4283 substances associated with plastic food packaging, and FCCdb contains 12 285 substances that could possibly be used worldwide to make FCMs. Searching for compounds with formulas derived from HRMS data in these two databases, rather than ChemSpider or PubChem, may reduce the list of potential candidates.

In this work, RT and CCS prediction models were developed using machine learning approaches and experimental values. Subsequently, the models were used to predict RT and CCS values of the substances in CPPdb and FCCdb. The two databases were then transformed into screening libraries and integrated into our structural elucidation workflow. The applicability of these two databases to the identification of FCCs was evaluated by studying the migration of compounds from PA spatulas. Chemicals that migrated from PA spatulas were screened against the CPPdb and FCCdb libraries and a plastic additives database developed in-house.

## Materials and Methods

### Chemicals and Reagents

A total of 675 standards containing antioxidants, plasticizers, photoinitiator, UV absorbers, slip agents, lubricants, degradation products of additives, and oligomers of polyethylene glycol (PEG) and polypropylene glycol (PPG) were purchased from Sigma-Aldrich Quimica S.A. (Madrid, Spain), Cymit Química S.L (Barcelona, Spain), Extrasynthese (Genay, France), and Cayman Chemical Company (Ann Arbor, MI). Oligomers of adhesives, polyamide (PA) and PLA, were isolated from the corresponding polymers using the procedures described in Canellas et al.^35^ HPLC grade ethanol (≥99.9%), methanol (≥99.9%), dichloromethane (≥99.8%), acetone (≥99.8%), and dimethyl sulfoxide (≥99.8%) were purchased from Scharlau Chemie S.A (Sentmenat, Spain). Deionized water was obtained from a Millipore Milli-QPLUS 185 system (Madrid, Spain).

Standard stock solutions of all standards were prepared in methanol at a concentration of 1000 mg/kg. For compounds not fully soluble in methanol, alternative solvents (ethanol, dichloromethane, acetone, and dimethyl sulfoxide) were used. Working solutions consisting of 8–10 analytes at approximately 1 mg/kg were prepared from the stock solutions. All solutions were kept at −20 °C until analyzed.

### Instrumentation

The standard solutions were analyzed using an Acquity I-Class UPLC system coupled to a Vion IMS-QTOF mass spectrometer with an electrospray ionization (ESI) source (Waters, Manchester, UK). Compounds were separated using a CORTECS C18 UPLC column (2.1 × 100 mm, 1.6 μm particle size, 90 Å pore size) with a flow rate of 0.3 mL/min. Gradient elution was performed using water (A) and methanol (B) as mobile phases, both containing 0.1% (v/v) formic acid. The initial percentage of mobile phase B was 5%, which linearly increased to 100% over 7 min. This was followed by a 4 min isocratic period, then returned to initial conditions from 11 to 11.1 min, and re-equilibrated at 5% B from 11.1 to 13 min. The sample and column temperatures were 10 and 40 °C, respectively, and the injection volume was 5 μL.

ESI was performed in both positive and negative ionization modes. The capillary voltage was 1 kV and the cone voltage 30 V, and the source temperature was set to 120 °C and the desolvation temperature to 500 °C. The cone gas flow rate was 50 L/h, and the desolvation gas flow was 800 L/h. Data were acquired in high-definition MS^E^ mode (HDMS^E^) over the *m*/*z* range 50–1000 Da. The instrument switched between two collision energies (low energy, 6 eV; high energy, ramp 20–40 eV) to obtain precursor and fragment ions in a single run. Leucine-enkephalin ([M + H]^+^, *m*/*z* 556.2766; [M – H]^−^, *m*/*z* 554.2620) at a concentration of 100 ng/mL was infused at a rate of 15 μL/min for real-time mass correction. The mass analyzer was operated with a resolution of around 40 000 full width half-maximum (fwhm) at *m*/*z* 556, and ion mobility resolution was ∼20 Ω/ΔΩ fwhm. The IMS gas flow rate was 25 mL/min with a wave velocity of 250 m/s and an IMS pulse height of 45 V. The Major Mix IMS/Tof calibration kit (ref. 186008113) from Waters (Manchester, UK) was used for CCS calibration. More information about standards injection, quality control, and precision of CCS measurement can be seen in a previous study.^26^ Data acquisition and processing were carried out using the UNIFI v.1.9.4 scientific information system (Waters, Manchester, UK). The Vion platform works at a room temperature of 25 °C.

### RT Prediction

The RT prediction was performed using the R package *Retip*.^28^ Three algorithms, Extreme Gradient Boosting (XGBoost), random forest (RF), and Bayesian regularized neural network (BRNN), were used for model building, and their prediction performances were compared.

The SMILES and InChIKey of each compound were retrieved from PubChem using the R package *webchem*.^36^ Chemistry Development Kit chemical descriptors of 675 compounds were then calculated; the returned data contained 667 compounds, as the descriptors of eight sodium-containing compounds were not successfully calculated. The descriptors with zero or low variance were eliminated, and 134 descriptors were retained. The data set was then randomly split into training and testing sets in the ratio of 8:2; 535 and 132 RT records were included in the training set and testing set, respectively. The model was built using the training set and validated using the testing set. More information about the use of *Retip* can be seen in the study from Bonini and co-workers.^28^

### CCS Prediction

The prediction of CCS values of chemicals associated with plastic products has been described in a previous study,^37^ in which the CCS prediction models were built using support vector machine regression (SVM) based on 1076 [M + H]^+^ CCS values and 645 [M + Na]^+^ CCS values. As CCS values are reproducible across different laboratories and platforms, some CCS records for model building were from other publications.^19,25,38−41^

### Prediction of RT and CCS Values of Substances in CPPdb and FCCdb

The rationalization of the substances in the CPPdb and FCCdb databases was performed in a previous study,^37^ where metals and salts, together with any substances with the same InChIKey (replicates), were removed. Additionally, compounds with a molecular weight outside the range 50–1200 Da were removed, leaving 2883 and 6508 substances in the CPPdb and FCCdb, respectively. Subsequently, RT and CCS values for the remaining compounds in CPPdb and FCCdb were predicted using the models described above. The two databases, together with the RT and CCS predictions, were converted into screening libraries to be used for suspect screening.

### Migration Test

Spatulas made from PA were purchased from a local market. The migration experiments conducted on the spatulas were described in a previous study.^35^ In summary, 95% (v/v) ethanol was used as a food simulant; the spatula was cut into 1 × 5 cm pieces and placed into glass vials filled with 41.6 g of simulant. The surface to volume ratio of 0.96 dm^2^/L was selected since it is the real surface to volume ratio for spatulas used for 1 L of food. BfR recommends that the migration test should be 100 °C for 0.5 h for PA kitchen utensils;^42^ however, evaporation of ethanol occurs using the migration temperature of 100 °C. To mitigate this situation, the vials were placed in an oven at 60 °C for 2.5 h. Migration tests were conducted in triplicate, and 95% ethanol was used as a blank.

### Identification Workflow

**Figure 1:**
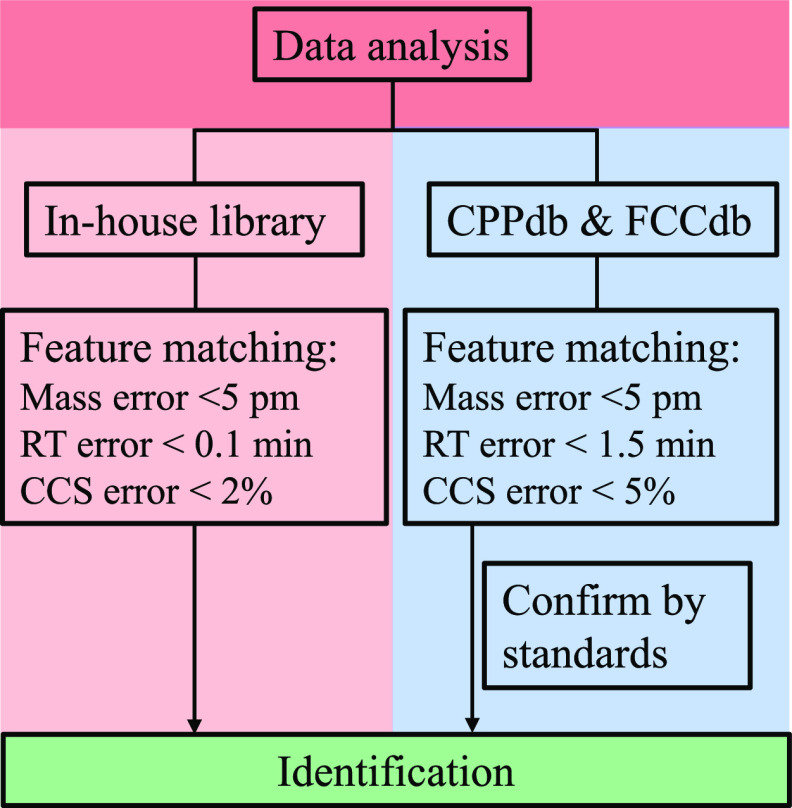
Workflow for the identification of migrants from a PA spatula.

The process shown in Figure 1 is used to identify compounds that migrate from the PA spatula. 675 standards, which included additives and NIAS commonly found in FCMs, were analyzed, and their *m*/*z* values, adducts, RT, CCS values, and fragment ions were added to an in-house database. The migration samples were screened against the in-house library with the criteria of *m*/*z* error <5 ppm, RT error <0.1 min, and CCS delta <2%.

Features (*m*/*z*_RT_CCS) that were not tentatively assigned to compounds in the in-house library were then screened against the two plastic packaging-related screening libraries created from the CPPdb (2883 compounds) and FCCdb (6508 compounds), with *m*/*z*, adducts, predicted RT, and CCS values. The screening tolerances in this case were based on the accuracy of the predicted values. For those compound that were tentatively assigned after screening against all three libraries, commercial standards were purchased, where possible, to confirm the identification.

## Results and Discussion

### RT and CCS Prediction

**Figure 2:**
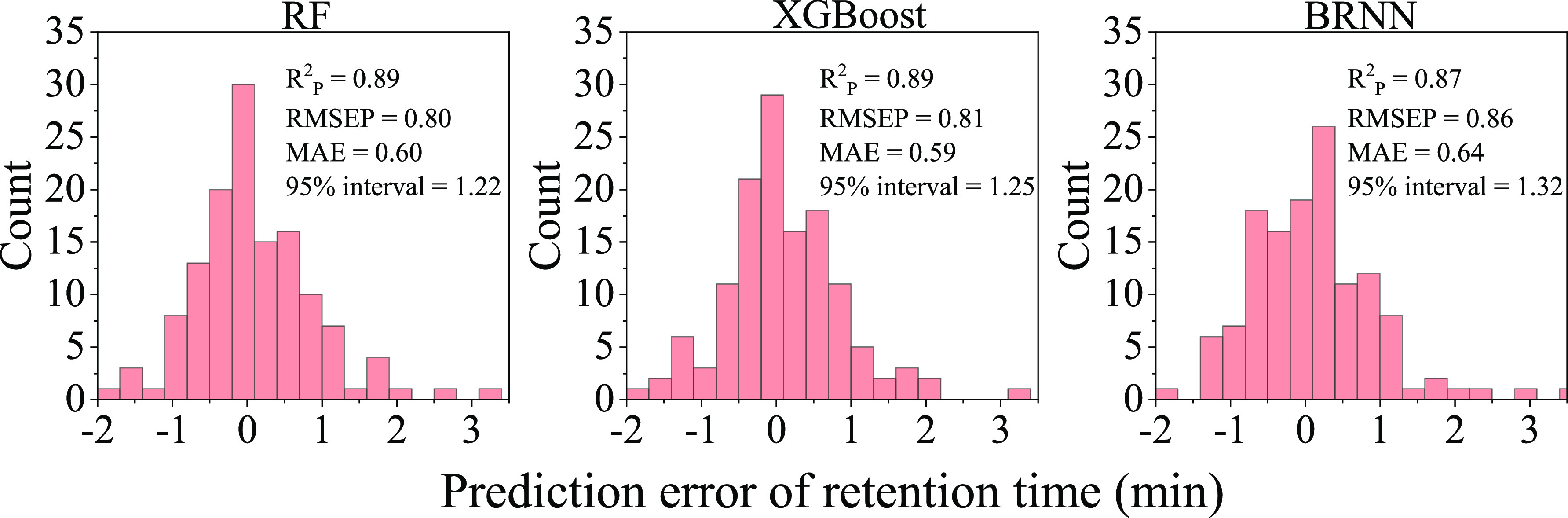
Histograms showing the prediction errors for retention time using the random forest (RF), extreme Gradient Boosting for tree algorithms (XGBoost), and Bayesian Regularized Neural Network (BRNN) models. *R*^2^_p_, external validation coefficient of determination; RMSEP, root-mean-square error of prediction; MAE, mean absolute error; 95% interval, prediction errors in the 95% confidence interval.

After splitting the data set into a training set and a testing set, 132 RT records were included in the testing set, and three algorithms were used to build RT prediction models. A comparison of the performance of the three algorithms is shown in Figure 2. The figure shows that RF and XGBoost have similar prediction capabilities, and both outperformed BRNN. The configuration of the RF algorithm involves fewer hyperparameters and is easier to tune than the XGBoost algorithm. Therefore, the RF algorithm was used to develop the RT prediction model and for predicting the RT values of the substances in the CPPdb and FCCdb libraries. Figure 2 shows that the RF-based model generated prediction errors within 1.22 min for the set of test compounds for a 95% confidence interval. Consequently, the RT tolerance to screen the measured data against the CPPdb and FCCdb was set to 1.5 min, slightly wider than the value of 1.22 min so as to not automatically discard true positives.

In the CCS prediction model, the CCS values of 93.3% of [M + H]^+^ adducts and 95.0% of [M + Na]^+^ adducts in the test data set were predicted with less than 5% error.^37^ Therefore, the tolerance used for the CCS values on screening the measured data against the CPPdb and FCCdb libraries was set to 5%.

### Identification of Migrating Compounds Using the In-House Library

A total of 51 compounds were identified upon searching against the in-house library. These included common additives such as plasticizers, antioxidant, slip agents, and lubricants. NIAS, typically oligomers and degradation products of additives, were also found in the PA spatulas. Detailed information on the identifications is provided in Table 1.

**Table 1:** Compounds That Have Migrated from a Polyamide (PA) Spatula Sample into 95% Ethanol Identified Using an In-House Plastics Additives Library

no.	RT_exp_ (min)	ΔRT (min)	CCS_exp_ (Å^2^)	ΔCCS (%)	observed *m*/*z*	*m*/*z* error (ppm)	fragments	adducts	molecular formula	candidate name	remarks
1	7.17	0.04	178.4	0.43	309.2036	0.0		[M + Na]^+^	C_16_H_30_O_4_	2,2,4-trimethyl-1,3-pentanediol diisobutyrate	plasticizer
2	7.74	0.09	191.4	–0.10	337.2351	0.4		[M + Na]^+^	C_18_H_34_O_4_	dibutyl sebacate	plasticizer
3	7.95	0.05	211.0	0.01	363.2502	–1.2	281.0510, 319.1945	[M + Na]^+^	C_20_H_36_O_4_	bis(2-ethylhexyl) maleate	plasticizer
4	8.20	0.07	219.0	0.35	413.2660	–0.5		[M + Na]^+^	C_24_H_38_O_4_	dioctyl phthalate/diisooctyl phthalate/bis(2-ethylhexyl) phthalate	plasticizer
5	8.22	0.07	221.3	1.08	393.2973	–0.6	147.0656	[M + Na]^+^	C_22_H_42_O_4_	bis(2-ethylhexyl) adipate	plasticizer
6	8.49	0.10	226.2	0	441.2975	0.0		[M + Na]^+^	C_26_H_42_O_4_	dinonyl phthalate/diisononyl phthalate	plasticizer
7	6.60	0.03	176.8	0.71	277.1808	–0.6	175.1127, 205.1595, 119.0502, 233.1908	[M – H]^−^	C_17_H_26_O_3_	3-(3,5-ditert-butyl-4-hydroxyphenyl)propanoic acid	degradation products
8	6.60	0.06	164.7	1.21	233.1544	–1.2	217.1235	[M – H]^−^	C_15_H_22_O_2_	3,5-ditert-butyl-4-hydroxybenzaldehyde	degradation products
9	6.66	0.04	169.6	0.44	247.1703	–0.3	96.9597	[M – H]^−^	C_16_H_24_O_2_	3,5-ditert-butyl-4-hydroxyacetophenone	degradation products
10	7.16	–0.09	241.5	–0.46	551.3845	–1.6		[M – H]^−^	C_34_H_52_N_2_O_4_	Irganox 1024	antioxidant
11	7.47	0.09	273.9	0.95	637.4941	0.4	304.2273, 377.3165	[M + H]^+^	C_40_H_64_N_2_O_4_	Irganox 1098	antioxidant
12	7.78	0.07	187.8	0.68	256.2635	0.1		[M + H]^+^	C_16_H_33_NO	hexadecanamide	slip agent
13	7.87	0.04	191.0	0.29	282.2794	1.0		[M + H]^+^	C_18_H_35_NO	oleamide	slip agent
14	7.93	–0.07	195.7	0.48	284.2947	–0.3		[M + H]^+^	C_18_H_37_NO	octadecanamide	slip agent
15	8.41	0.10	202.6	0.42	360.3239	0.5		[M + Na]^+^	C_22_H_43_NO	erucamide	slip agent
16	7.80	0.01	209.0	1.00	392.3121	–3.5		[M + Na]^+^	C_22_H_43_NO_3_	*N*,*N*-diethanololeamide	antistatic agent
17	8.03	0.08	201.7	0.56	365.2661	–0.5		[M + Na]^+^	C_20_H_38_O_4_	glycol ricinoleate	antistatic agent
18	8.13	0.06	172.8	0.30	317.2458	2.1		[M + Na]^+^	C_19_H_34_O_2_	9,12-octadecadienoic acid, methyl ester	antistatic agent
19	8.14	0.07	206.1	1.15	381.2974	–0.5	282.0518	[M + Na]^+^	C_21_H_42_O_4_	glyceryl monostearate	antistatic agent
20	7.36	0.07	161.4	1.04	199.1702	–0.6		[M – H]^−^	C_12_H_24_O_2_	lauric acid	lubricant
21	8.08	0.08	176.3	0.68	255.2329	–0.1		[M – H]^−^	C_16_H_32_O_2_	palmitic acid	lubricant
22	8.38	0.10	184.5	–0.80	283.2643	0.2	265.2535	[M – H]^−^	C_18_H_36_O_2_	stearic acid	lubricant
23	11.73	0.01	280.9	0.68	615.5811	1.9		[M + Na]^+^	C_38_H_76_N_2_O_2_	*N*,*N*′-ethylenebis(stearamide)	lubricant
24	3.17	0.04	181.5	–0.09	362.2415	0.3	114.0914, 227.1753	[M + Na]^+^	C_18_H_33_N_3_O_3_	1,8,15-triazacyclohenicosane-2,9,16-trione	PA6 trimer
25	3.67	0	210.9	1.21	453.3432	–0.8	114.0908, 209.1646	[M + H]^+^	C_24_H_44_N_4_O_4_	1,8,15,22-tetraazacyclooctacosane-2,9,16,23-tetrone	PA6 tetramer
26	4.10	0.04	233.7	0.19	588.4090	–0.9	114.0911, 435.3328	[M + Na]^+^	C_30_H_55_N_5_O_5_	1,8,15,22,29-pentazacyclopentatriacontane-2,9,16,23,30-pentone	PA6 pentamer
27	4.43	0.08	265.5	1.21	701.4935	–0.1	209.1647, 548.4169	[M + Na]^+^	C_36_H_66_N_6_O_6_	1,8,15,22,29,36-hexazacyclodotetracontane-2,9,16,23,30,37-hexone	PA6 hexamer
28	2.70	0.09	152.0	0.49	227.1753	–0.5	100.1119, 209.1647	[M + H]^+^	C_12_H_22_N_2_O_2_	1,8-diazacyclotetradecane-2,7-dione	PA66 monomer
29	3.92	0.04	211.0	0.19	453.3432	–0.8	100.1119, 182.1535, 209.1644, 326.2800	[M + H]^+^	C_24_H_44_N_4_O_4_	1,8,15,22-tetraazacyclooctacosane-2,7,16,21-tetrone	PA66 dimer
30	4.61	0.06	266.2	–0.28	701.4939	0.4	182.1537, 552.4489	[M + Na]^+^	C_36_H_66_N_6_O_6_	1,8,15,22,29,36-hexaazacyclodotetracontane-2,7,16,21,30,35-hexone	PA66 trimer
31	2.32	0.03	150.9	0.47	261.1306	–1.1		[M + Na]^+^	C_10_H_22_O_6_	PEG5	PEG oligomer
32	2.61	0.01	160.1	1.25	305.1569	–0.6		[M + Na]^+^	C_12_H_26_O_7_	PEG6	PEG oligomer
33	2.87	0.02	167.0	1.08	349.1833	0.0		[M + Na]^+^	C_14_H_30_O_8_	PEG7	PEG oligomer
34	3.08	0	178.6	1.40	393.2095	0.0		[M + Na]^+^	C_16_H_34_O_9_	PEG8	PEG oligomer
35	3.28	0.01	188.4	0.23	437.2355	–0.6	182.1539, 394.2310	[M + Na]^+^	C_18_H_38_O_10_	PEG9	PEG oligomer
36	3.44	0	197.3	0.16	481.2616	–0.7		[M + Na]^+^	C_20_H_42_O_11_	PEG10	PEG oligomer
37	3.59	0	207.3	1.37	525.2877	–0.8	182.1536, 226.1910	[M + Na]^+^	C_22_H_46_O_12_	PEG11	PEG oligomer
38	3.72	0	220.5	1.77	569.3147	0.7		[M + Na]^+^	C_24_H_50_O_13_	PEG12	PEG oligomer
39	4.72	–0.03	173.8	1.48	331.2088	–0.8		[M + Na]^+^	C_15_H_32_O_6_	PPG5	PPG oligomer
40	5.24	–0.03	185.9	0.15	389.2504	–1.6		[M + Na]^+^	C_18_H_38_O_7_	PPG6	PPG oligomer
41	5.66	–0.03	198.5	–0.34	447.2927	–0.4	399.2615	[M + Na]^+^	C_21_H_44_O_8_	PPG7	PPG oligomer
42	5.98	–0.05	211.1	0.60	505.3343	–0.8	475.3258	[M + Na]^+^	C_24_H_50_O_9_	PPG8	PPG oligomer
43	6.28	–0.02	224.6	1.49	563.3756	–1.7	175.1327	[M + Na]^+^	C_27_H_56_O_10_	PPG9	PPG oligomer
44	6.51	–0.03	238.2	1.73	621.4179	–0.8		[M + Na]^+^	C_30_H_62_O_11_	PPG10	PPG oligomer
45	6.71	–0.03	253.2	1.65	679.4592	–1.6	592.4079, 619.4069	[M + Na]^+^	C_33_H_68_O_12_	PPG11	PPG oligomer
46	5.29	0.05	141.1	0.61	179.0711	–1.3		[M – H]^−^	C_10_H_12_O_3_	propylparaben	biocide
47	5.58	0.08	161.9	–1.05	242.1761	–0.2	181.1598	[M + HCOO]^−^	C_12_H_23_NO	12-aminododecanolactam	monomer
48	5.97	–0.10	184.2	–0.02	355.1457	4.6		[M + H]^+^	C_23_H_18_N_2_O_2_	2-diphenylacetyl-1,3-indandione-1-hydrazone	
49	7.29	0.06	203.4	–0.62	379.1700	–0.5	196.0888	[M + H]^+^	C_21_H_28_Cl_2_N_2_	4,4′-methylenebis(3-chloro-2,6-diethylaniline)	curing agent
50	7.48	0.07	233.5	0.06	507.2709	–1.6		[M + Na]^+^	C_29_H_40_O_6_	1,2,3-trideoxy-4,6:5,7-bis-*O*-((4-propylphenyl)methylene)-nonitol	nucleating agent
51	8.87	0.10	228.9	1.02	431.1788	0.0	415.1477	[M + H]^+^	C_26_H_26_N_2_O_2_S	2,5-bis(5-*tert*-butyl-2-benzoxazolyl)thiophene	brightener

Among these additives, six plasticizers were found in the PA spatula, including the structures of a fatty acid ester and phthalates. It should be noted that the identities of phthalate-based plasticizers were not confirmed due to the presence of structural isomers. For example, the [M + Na]^+^ ions of three isomers, dioctyl phthalate, diisooctyl phthalate, and bis(2-ethylhexyl) phthalate, showed good agreement with a component of *m*/*z* 413.2660, RT 8.20 min, and CCS 219.0 Å^2^. The RT and CCS values for dioctyl phthalate, diisooctyl phthalate, and bis(2-ethylhexyl) phthalate in the library are 8.13 min and 218.3 Å^2^, 8.10 min and 217.9 Å^2^, and 8.19 min and 217.5 Å^2^, respectively. As such, the RT and CCS values of all three compounds were within the screening tolerances of 0.1 min and 2%, respectively.

Antioxidants are commonly used as additives in plastics to prevent the oxidation of polymers during processing, storage, and usage; two hindered phenolic antioxidants, Irganox 1024 and Irganox 1098, were found in the PA spatula. Additionally, three degradation products, 3-(3,5-di-*tert*-butyl-4-hydroxyphenyl)propanoic acid, 3,5-di-*tert*-butyl-4-hydroxybenzaldehyde, and 3,5-di-*tert*-butyl-4-hydroxyacetophenone, were also identified. These degradation products can originate from the oxidation of Irganox 1024 and Irganox 1098 and have been found in other plastic products.^14,43^

The fatty amides oleamide and erucamide, and fatty acids palmitic acid and stearic acid, were identified in the PA spatula. Compounds such as these are commonly used as slip agents and lubricants in plastics.^44^ The slip agents oleamide and erucamide can form a microcrystalline structure on the surface of films, thereby reducing the friction coefficient of the films.^45^ The antistatic agent *N*,*N*-diethanololeamide and glycerin derivatives, such as glyceryl monostearate, were also detected in the PA spatula.

**Figure 3:**
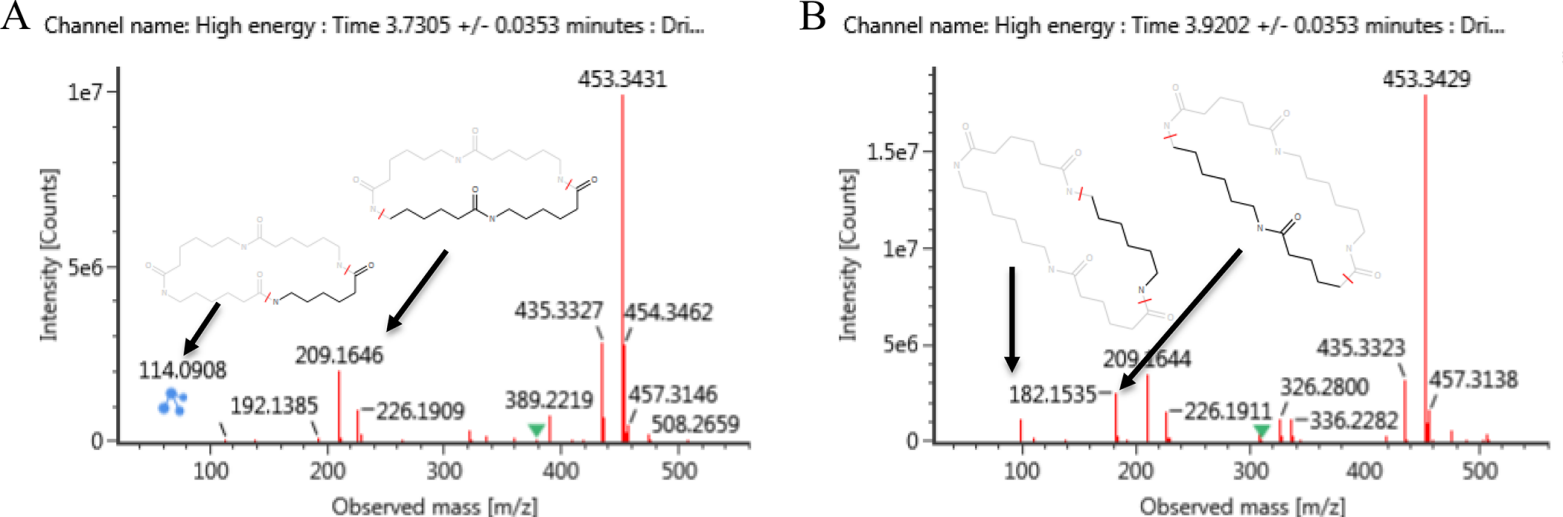
High-energy spectra of the PA6 tetramer (A) and PA66 dimer (B).

The most abundant group of compounds detected in the PA spatula was PA oligomers, including four PA6 oligomers and three PA66 oligomers. PA6 is a polymer of ε-caprolactam, and PA66 is a polymer of 1,6-hexanediamine and adipic acid. The PA6 tetramer 1,8,15,22-tetraazacyclooctacosane-2,9,16,23-tetrone and PA66 dimer 1,8,15,22-tetraazacyclooctacosane-2,7,16,21-tetrone have identical molecular formulas and have similar CCS values; however, they do have different RTs with the PA6 tetramer eluting earlier than PA66 dimer. We compared the high-energy spectra of the PA6 tetramer and PA66 dimer to ascertain whether there are different fragment ions for these two compounds. It can be seen in Figure 3 that the two compounds have common fragment ions at *m*/*z* 209.1646, 226.1909, and 435.3327. A characteristic fragment ion with *m*/*z* 114.0908 is observed for the PA6 tetramer which corresponds to the *m*/*z* of caprolactam, the monomer of PA6. The PA66 dimer has characteristic fragment ions with *m*/*z* 100.1118, 111.0436, 128.0703, 182.1535, and 326.2800. The fragment ion at *m*/*z* 100.1118 can be explained by 1,6-hexanediamine losing NH_2_, and the fragment ion at *m*/*z* 111.0436 can be explained by adipic acid losing two hydroxyl groups. The structures corresponding to fragment ions at *m*/*z* 128.0703 and 326.2800 can see seen in Figure S1. The unique fragments for each isomer provide valuable information that helps to distinguish between PA6 and PA66 oligomers.

Eight polyethylene glycol (PEG) oligomers and seven polypropylene glycol (PPG) oligomers were found in the PA spatula which is consistent with the oligomers being detected in other PA kitchenware.^46^ These oligomers are highly viscous and are usually blended with lower-viscosity plasticizers in plastics. It has been reported that the grafting of PEG on polyvinyl chloride (PVC) surface reduces the diffusion of bis(2-ethylhexyl) phthalate from the PVC matrix. This is possibly due to the high hydrophilicity of PEG.^47^ Plotting the CCS values against the *m*/*z* values for the PEG and PPG oligomers reveals a high linear correlation, as shown in Figure S2, with an *R*^*2*^ of 0.9907. PEG and PPG oligomers tend to form compact structures due to intramolecular hydrogen bonds;^48^ thus, their CCS values are relatively lower when compared to other compounds with similar *m*/*z* values.

Figure S3 shows the mass spectra of PPG5 both with and without drift time (DT) alignment. As precursor and fragment ions always share the same DT, the alignment based on both RT and DT enables many interfering ions to be eliminated, producing cleaner mass spectra. This is a big advantage of using IMS-HRMS in targeted and suspect screening analyses. Figure S3B shows that PPG5 in fact exhibits no fragmentation, which could be due to the low concentration, or rigid structure of the analyte. The use of IMS-QTOF gives higher confidence to the identification of compounds when no fragment ions are present, as it provides CCS as an additional identification point in addition to *m*/*z* and RT.

Despite these benefits of IMS in targeted and suspect screening analysis, crucial information provided by LC cannot be ignored. For example, the isomeric pair of the PA6 tetramer (CCS = 210.9 Å^2^, RT = 3.73 min) and the PA66 dimer (CCS = 211.0 Å^2^, RT = 3.92 min) have similar experimental CCS values, and their identifications were confirmed due to their different RT values. The power of LC-IMS-HRMS originates from the multidimensional structural information that this technique can provide.

### Identification of Migrating Compounds Using the CPPdb and FCCdb Libraries

**Figure 4:**
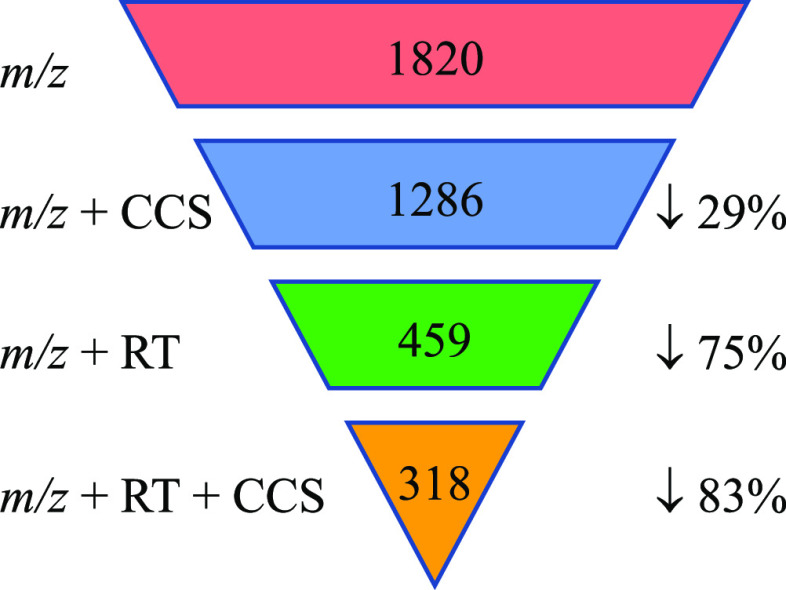
Number of candidates retained on applying different filters. *m*/*z* filter is 5 ppm, CCS filter 5%, and RT filter 1.5 min.

The components detected in the PA spatula migration samples were screened against the 9391 compounds in the CPPdb and FCCdb library. The effect of filtering library matches using RT and CCS, and a combination of the two, on the number of possible candidates is shown in Figure 4. Using the RT filters alone eliminates more false positives from the list of candidates than using the CCS filter alone. Approximately 75% (1361 out of 1820) of the candidates were excluded as likely false positives using an RT tolerance of 1.5 min. A similar reduction in the number of candidates was observed by Bonini et al.,^28^ with an average of 68% of all candidate eliminated using an RT tolerance of 1 min. Using the CCS filter alone resulted in a reduction of 29% (534 out of 1820) of candidates. This is comparable with the reduction reported in the study of Bijlsma et al.,^49^ in which 9–39% of candidates were excluded by applying a CCS tolerance of 6%. The fact that fewer false positives are eliminated using the CCS filter may be because CCS is highly correlated to the molecular weight (MW) of the compound.^20,21,26^ RT, on the contrary, determined mostly by the octanol/water partition coefficient,^28^ and showing a lower correlation to MW (see Figure S4), provides complementary structural information in screening applications.

It should be noted that a feature does not mean a chemical; many of the features could be system noises or in-source fragments.^50^ Thus, the 318 features retained after applying *m*/*z*, RT, and CCS matching were further checked manually, in order to eliminate the features showing similar responses in the blank reference (95% ethanol), as well as the features having a low response that are not possibly precursor ions. Finally, a total of 44 compounds were tentatively identified on screening the measured data against the CPPdb and FCCdb libraries with respect to RT, CCS, and *m*/*z*. Detailed information, including RT, CCS, adducts, molecular formula, and mass error, are summarized in Table S1.

Three alkyl PEG ethers were identified, namely, polidocanol, ceteth-3 (emulsifying), and Arosurf. Compounds of this type, which are also called alkyl ethoxylates, are commonly used as nonionic surfactants in plastics.^51^ Tisler and co-workers found that alkyl PEG ethers migrated from reusable polyethylene (PE) water bottles.^52^ The RT of alkyl PEG ethers is dependent on the number of ethoxylate (EO) groups in the molecule. Each EO group in the molecular chain increases the polarity of the molecule; therefore, the more EO groups present in an alkyl PEG ether, the earlier the chromatographic elution of the compound in reverse phase LC. This was demonstrated by the elution order of polidocanol (7.59 min), ceteth-3 (8.16 min), and Arosurf (8.61 min), which contain 9, 3, and 2 EO groups in their molecular structures, respectively. Three derivatives of alkyl PEG ethers were also identified, 2-(2-(2-(dodecyloxy)ethoxy)ethoxy)ethyl hydrogen maleate, 3,6,9,12-tetraoxatetracosan-1-ol dihydrogen phosphate, and octaethylene glycol laurate. 2-(2-(2-(Dodecyloxy)ethoxy)ethoxy)ethyl hydrogen maleate can form in the reaction between triethylene glycol monododecyl ether and maleic acid. 3,6,9,12-Tetraoxatetracosan-1-ol dihydrogen phosphate can be produced by the esterification between tetraethylene glycol monododecyl ether and phosphoric acid. Octaethylene glycol laurate can form in the reaction between octaethylene glycol and lauric acid. All three of these derivatives can also be used as surfactants.

**Figure 5:**
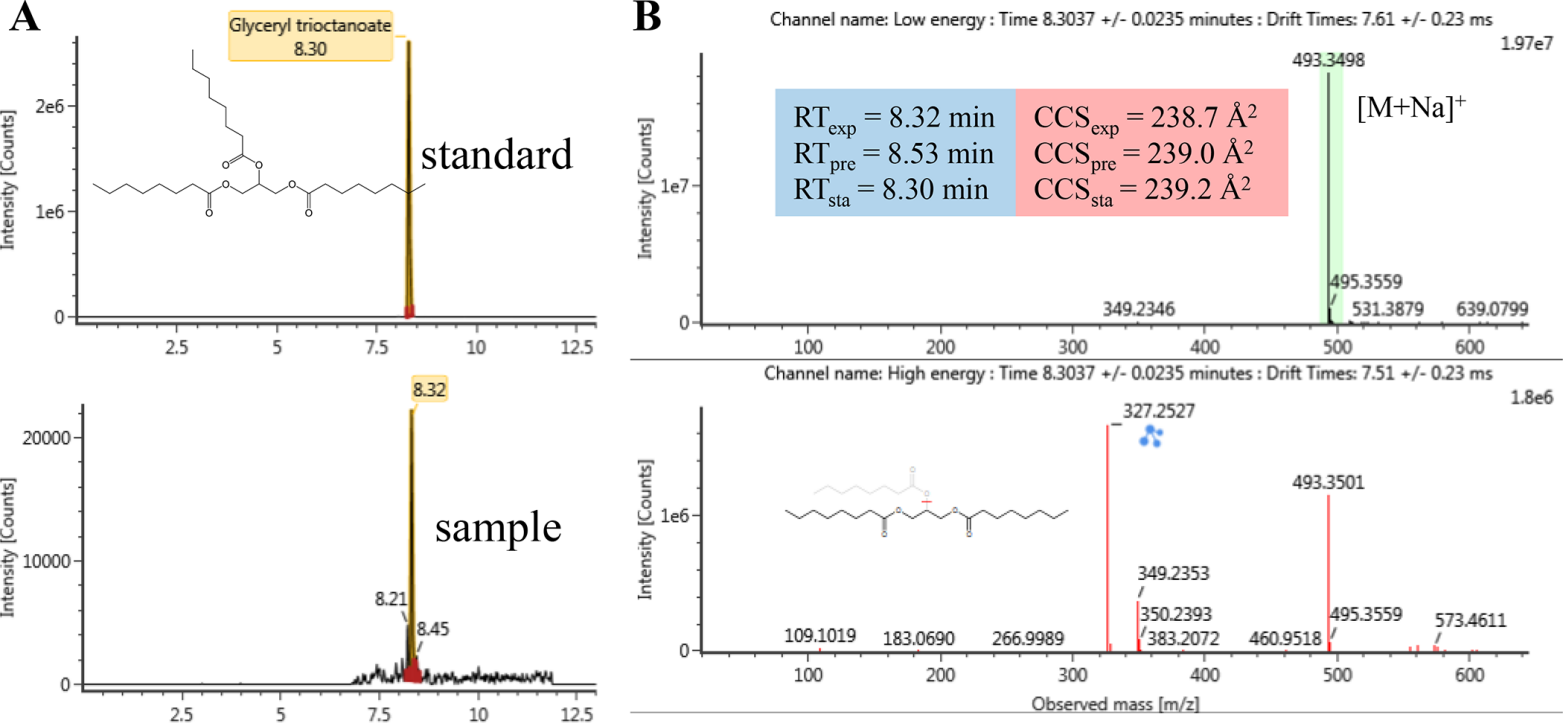
Identification of glyceryl trioctanoate. (A) Extracted ion chromatograms for the [M + Na]^+^ adduct (*m*/*z* 493.3498). (B) Low- and high-energy spectra, fragment assignments, and a comparison between experimental and predicted RT and CCS values.

Five antistatic agents were tentatively identified, a sorbitan fatty acid ester (sorbitan laurate), a glycol derivative (glycol oleate), and the following three glycerol derivatives: decanoic acid ester with 1,2,3-propanetriol octanoate; glyceryl trioctanoate; and C16–18 mono- and diglycerides. Antistatic agents are also types of surfactants with the polyol amine (*N*,*N*-diethanololeamide), sorbitan laurate, glycol, and glycerol derivatives having moisturizing properties that are capable of forming a film of water on the surface of plastics to prevent static electricity from occurring. The identification of glyceryl trioctanoate was further confirmed by measuring the reference standard and comparing extracted ion chromatograms, experimental and predicted RT and CCS values, mass spectra, and fragment assignments, as shown in Figure 5. The fragment ion with an *m*/*z* value of 327.2527 [M – C_8_H_15_O_2_]^+^ can be derived from the loss of an octanoate anion. The fragment ion with an *m*/*z* value of 349.2353 has a mass difference of 21.9826 from 327.2527, corresponding to [M + Na – C_8_H_15_O_2_]^+^. The predicted RT and CCS values show a good match with experimental values (ΔRT = 0.21 min and ΔCCS = 0.3 Å^2^), and therefore, using predicted RT and CCS values gave higher confidence in the identification.

Four plasticizers were tentatively identified using the CPPdb and FCCdb libraries, namely, bis(2-butoxyethyl) adipate; pentanedioic acid, bis(2-(2-(2-butoxyethoxy)ethoxy)ethyl) ester; ditridecyl adipate; and tris(2-ethylhexyl) trimellitate. The identity of tris(2-ethylhexyl) trimellitate was confirmed by comparing the measured data to that for a reference standard. Additionally, this compound has also been previously detected in PA kitchenware.^46^ The lubricants hexadecanamide, *N*,*N*′-1,2-ethanediylbis, and ethylene-*N*-palmitamide-*N*′-stearamide were found in the migration samples from the PA spatula; the former has been detected in PE films by Vera and co-workers.^44^

The organophosphate flame retardant triphenyl phosphate was identified in the PA spatula sample and confirmed using a reference standard. Chromatograms, mass spectra, fragment ions, and predicted RT and CCS values for triphenyl phosphate are shown in Figure S5. The fragment ion with *m*/*z* value 251.0466 indicates the loss of a phenyl group while the fragment ion with *m*/*z* value 77.0385 corresponded to the retained benzene. Fragment ions with *m*/*z* values of 152.0618, 168.0569, and 215.0255 are also present in mass spectra from MassBank of North America (MoNA). The experimental and predicted CCS values of the [M + H]^+^ adduct matched well, with ΔCCS% = 0.1%. The predicted CCS value for the [M + Na]^+^ adduct of triphenyl phosphate was not as accurate, with experimental and predicted CCS values of 181.8 and 187.8 Å^2^, respectively, corresponding to a CCS deviation of 3.3%. However, this value is still below the screening tolerance of 5%.

2-Ethylhexyl 4-(dimethylamino)benzoate, which can be used as a photoinitiator, was also identified in the sample together with the dimer 6-(6-aminohexanamido)hexanoic acid which can result from the polymerization of two 6-aminohexanoic acids. It should be noted that 6-aminohexanoic acid can be used to produce caprolactam. Finally, the fatty acid ester methyl 9,10-dihydroxyoctadecanoate was identified which may result from the oxidation of methyl oleate.

A limitation of using predicted RT and CCS values is that false positives identifications can occur. As an example of this, the features with *m*/*z* 553.3973, RT 6.85 min, and CCS 234.4 Å^2^ and *m*/*z* 665.3831, RT 7.06 min, and CCS 260.8 Å^2^ matched Irganox 1024 and Irganox 1035, respectively, under the screening conditions (RT error <1.5 min and ΔCCS% < 5%). However, upon measuring the reference standards of these compounds, it was found that the identifications were incorrect, as the RT of standards of Irganox 1024 and Irganox 1035 are 7.22 and 7.96 min, respectively. These features could be from other compounds that show a similar molecular weight. This means that although the compounds in Table S1 met all of the identification criteria, the assignments can only be completely confirmed using reference standards. Therefore, the addition of predicted RT and CCS values into the identification process can improve identification confidence but do not confirm the presence of the compound unequivocally.

### Approaches to Improve the Identification of FCCs

The use of the CPPdb and FCCdb libraries in the untargeted screening of FCCs can significantly reduce the number of candidate compounds to be considered as potential matches to a component in the measured data. For example, bisphenol A bis(2-hydroxypropyl) ether, a common bisphenol derivative in plastic products, was identified in the PA spatula data by searching against the CPPdb and FCCdb libraries. However, if a search on the molecular formula of bisphenol A bis(2-hydroxypropyl) ether (C_21_H_28_O_4_) is performed in PubChem, over 3000 hits are returned, and the compounds listed first are steroids which are unlikely to appear in plastic packaging. Therefore, we can confidently state that using plastic-related or FCM-related databases in the identification process of FCCs can significantly reduce the number of false positives and improve the confidence of identifications.

A limitation of using the CPPdb and FCCdb libraries is that although they currently contain 4283 and 12 285 substances, respectively, there are emerging substances associated with FCMs that are not included in either of the databases, for example, PA, PEG oligomers, and PPG oligomers. With the rapid growth of novel FCCs, the two databases need to be continually expanded and updated.

The improvement of IMS resolving power and CCS repeatability would also help in the identification of FCCs. At present, it is difficult to differentiate between structural isomers of some phthalate-based plasticizers (for example, dioctyl phthalate, diisooctyl phthalate, and bis(2-ethylhexyl) phthalate), because their CCS values are all within 2% of each other. Reproducible CCS measurements within a 0.5% tolerance would allow differentiation between such isomers. The current TWIMS system has an IM resolution of 40–50;^53^ to efficiently separate these phthalate-based isomers would require an IM resolution of 200 or more. Recently, it has been reported that state-of-the-art ion mobility platforms, structures for lossless ion manipulations (SLIM)^54^ and cyclic IMS,^55^ are able to provide a resolving power >300, with low CCS measurement deviations <0.5%. These two techniques are promising to differentiate the structural isomers of plasticizers.

In summary, 51 compounds were identified in the PA spatula samples screening against the in-house plastic additives library, and an additional 44 compounds were identified by screening against compounds in two public FCC-related databases (CPPdb and FCCdb) enhanced with predicted RT and CCS values. The most abundant compounds in the migration samples were PA6 and PA66 oligomers, but a range of other additives, including plasticizers, slip agents, and antistatic agents, were also detected. Predicted RT and CCS values can be used effectively to reduce the number of false positives and increase confidence in the identifications. The accuracy of RT and CCS predictions can be improved by incorporating additional measured values in the relevant training sets.

## Supporting Information Available

Fragments of PA6 tetramer and PA66 dimer; CCS values vs *m*/*z* values for PEG and PPG oligomers; mass spectra of PPG5; retention time versus molecular weight; identification of triphenyl phosphate; and migrates identified by CPPdb and FCCdb (PDF)

## Abbreviations Used

PA: polyamide
IMS: ion mobility spectroscopy
QTOF: quadrupole time-of-flight
RT: retention time
CCS: collision cross section
FCCs: food contact chemicals
FCM: food contact materials
NIAS: nonintentionally added substance
PLA: polylactic acid
PET: polyethylene terephthalate
PU: polyurethane
HRMS: high-resolution mass spectrometer
TOF: time-of-flight
RSDs: relative standard deviations
CPPdb: Chemicals associated with Plastic Packaging Database
FCCdb: Food Contact Chemicals Database
PEG: polyethylene glycol
PPG: polypropylene glycol
ESI: electrospray ionization
HDMS^E^: high-definition MS^E^ mode
XGBoost: extreme gradient boosting
RF: random forest
BRNN: Bayesian regularized neural network
SVM: support vector machine regression
*R*^*2*^_*p*_: external validation coefficient of determination
RMSEP: root-mean-square error of prediction
MAE: mean absolute error
PVC: polyvinyl chloride
PE: polyethylene
EO: ethoxylate
MoNA: MassBank of North America
SLIM: structures for lossless ion manipulations
