# Supplementary material for: Identification of Nonvolatile Migrates from Food Contact Materials Using Ion Mobility–High-Resolution Mass Spectrometry and in Silico Prediction Tools
Source: J Agric Food Chem. 2022 Jul 20;70(30):9499–508. doi: 10.1021/acs.jafc.2c03615 (PMC9354260; doi:10.1021/acs.jafc.2c03615)
Supplement: Supplementary file 1 — jf2c03615_si_001.pdf [file jf2c03615_si_001.pdf]

Supporting Information

Identification of Non-Volatile Migrates from Food Contact Materials using Ion Mobility−High Resolution Mass Spectrometry and In-Silico Prediction Tools

Xue-Chao Song[1], Elena Canellas[1], Nicola Dreolin[2], Jeff Goshawk[2], Cristina Nerin[1,*]

1. Department of Analytical Chemistry, Aragon Institute of Engineering Research I3A, CPS-University of Zaragoza, Maria de Luna 3, 50018, Zaragoza, Spain.

2. Waters Corporation, Altrincham Road, SK9 4AX, Wilmslow, United Kingdom.

* Corresponding author: Cristina Nerin, Phone: +34 976761873, Email: cnerin@unizar.es

Table of Contents:

Figure S1. The structures corresponding to characteristic fragments of PA6 tetramer and PA66 dimer.

Figure S2. ${}^{TW}CCS_{N2}$ values vs *m/z* values for PEG and PPG oligomers.

Figure S3. Comparison of mass spectra of PPG5 ([M+Na]+, m/z 331.2088) (A) without drift time alignment and (B) with drift time alignment.

Figure S4. Retention time (RT) versus molecular weight (Da) for 667 compounds in the database.

Figure S5. Identification of triphenyl phosphate. (A) extracted ion chromatograms from standard and sample injection, (B) low and high energy mass spectra, fragment assignment, comparison between experimental and predicted RT and CCS values for its $[M+H]^+$ adduct.

Table S1. Compounds that have migrated from a polyamide (PA) spatula sample into 95% ethanol identified using the CPPdb and FCCdb libraries.

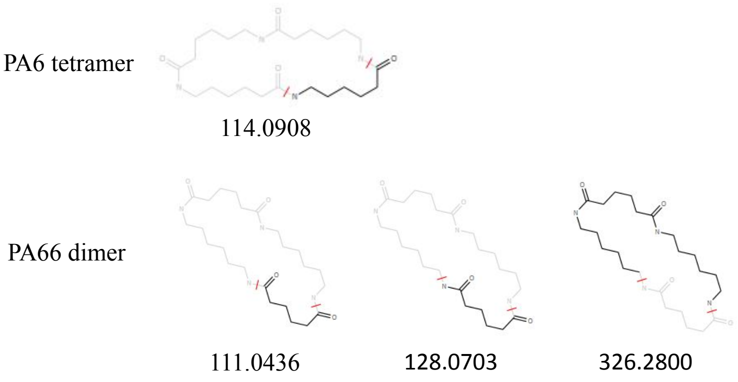

Figure S1. The structures corresponding to characteristic fragments of PA6 tetramer and PA66 dimer.

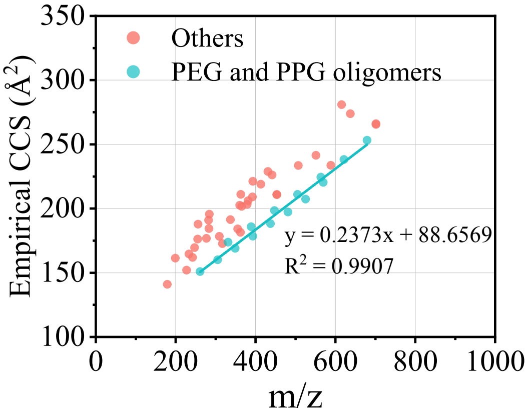

Figure S2. $^{TW}CCS_{N2}$ values vs *m/z* values for PEG and PPG oligomers.

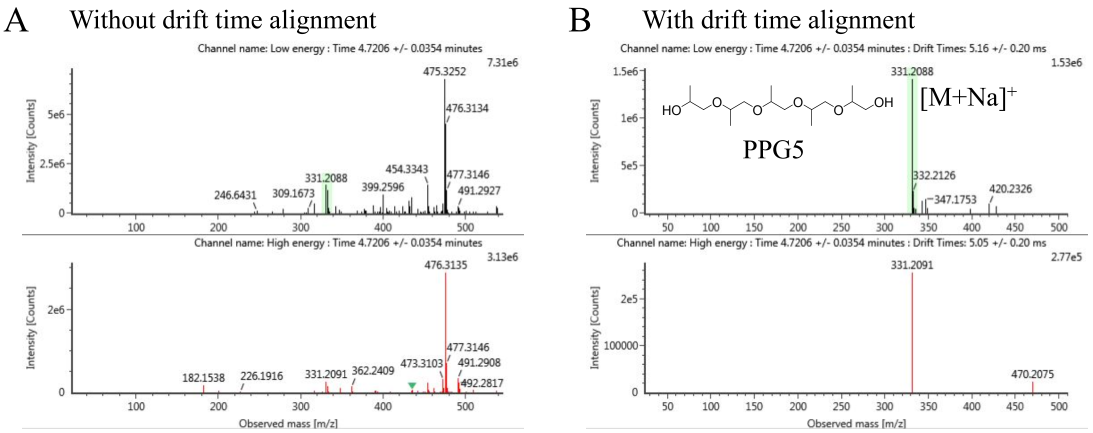

Figure S3. Comparison of mass spectra of PPG5 ([M+Na]$^+$, *m/z* 331.2088) (A) without drift time alignment and (B) with drift time alignment.

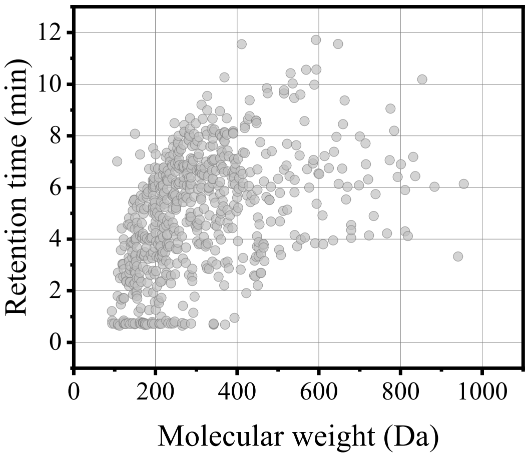

Figure S4. Retention time (RT) versus molecular weight (Da) for 667 compounds in the database.

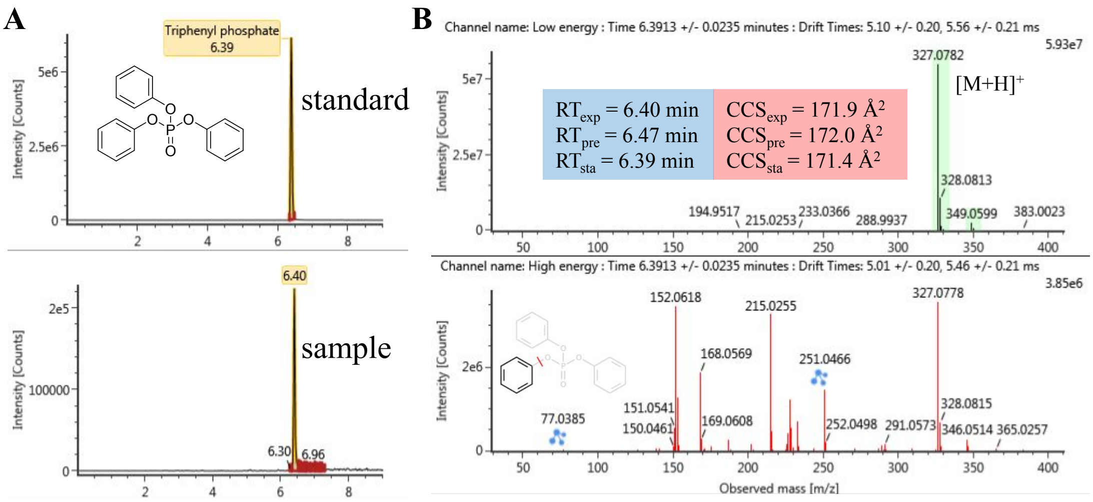

Figure S5. Identification of triphenyl phosphate. (A) extracted ion chromatograms from standard and sample injection, (B) low and high energy mass spectra, fragment assignment, comparison between experimental and predicted RT and CCS values for its $[M+H]^+$ adduct.

Table S1. Compounds that have migrated from a polyamide (PA) spatula sample into 95% ethanol identified using the CPPdb and FCCdb libraries.

No.	RT_{exp} (min)	ΔRT (min)	CCS_{exp} (Å^2)	ΔCCS (%)	Observed *m/z*	*m/z* error (ppm)	Adducts	Molecular formula	Compound name	PubChem CID	Remarks
1	0.84	-0.82	141.8	-0.78	217.1042	-0.6	$[M+H]^+$	$C_6H_{12}N_6O_3$	Trimethylolmelamine	70549	FCCdb
2	2.03	-0.68	166.6	0.76	245.1861	0.6	$[M+H]^+$	$C_{12}H_{24}N_2O_3$	6-(6-Aminohexanamido)hexanoic acid	895	CPPdb
3	2.26	-0.59	137.5	-3.36	190.1077	1.9	$[M+H]^+$	$C_8H_{15}NO_4$	Diethyl iminodiacetate	80502	FCCdb
4	2.68	0.59	150.9	-0.22	209.1647	-0.6	$[M+H]^+$	$C_{12}H_{20}N_2O$	Bis[(dimethylamino)methyl]phenol	3018067	FCCdb
5	3.67	0.05	149.1	-0.10	210.1488	-0.1	$[M+H]^+$	$C_{12}H_{19}NO_2$	N,N-Bis(2-hydroxypropyl)aniline	76498	FCCdb
6	4.45	0.47	137.0	-0.48	172.1117	-2.4	$[M+H]^+$	$C_{12}H_{13}N$	(S)-(-)-1-(1-Naphthyl)ethylamine	66325	FCCdb
7	4.61	0.83	160.1	1.85	228.1594	-0.1	$[M+Na]^+$	$C_{12}H_{21}NO_3$	4-[(2-Ethylhexyl)amino]-4-oxoisocrotonic acid	6913277	FCCdb
8	4.75	1.29	183.8	-3.88	399.2596	1.8	$[M+H]^+$	$C_{18}H_{38}O_9$	Oxirane, 2-methyl-, polymer with oxirane, ether with 1,2,3-propanetriol (3:1)	86278135	FCCdb
9	5.12	-1.23	199.4	-4.43	426.2681	-5.0	$[M+H]^+$	$C_{18}H_{43}NO_6Si_2$	Bis(3-(triethoxysilyl)propyl)amine	83535	FCCdb
10	5.6	-0.66	186.1	-2.88	369.2246	-0.4	$[M+Na]^+$	$C_{18}H_{34}O_6$	Sorbitan laurate	347468	FCCdb
11	5.72	-0.31	156.6	0.06	225.1485	-0.2	$[M+H]^+$	$C_{13}H_{20}O_3$	Isoamyl 4-(2-furan)butyrate	68968644	FCCdb
12	5.84	-0.91	169.1	-2.07	291.1566	-0.3	$[M+Na]^+$	$C_{15}H_{24}O_4$	1,9-Nonanediol diacrylate	9795378	FCCdb
13	5.95	-0.07	153.3	1.62	209.1538	1.1	$[M+H]^+$	$C_{13}H_{20}O_2$	(2-(1-Propoxyethoxy)ethyl)benzene	61403	FCCdb
14	6.07	0.59	151.9	0.86	211.1693	0.1	$[M+H]^+$	$C_{13}H_{22}O_2$	2-Bornyl propionate	89306	FCCdb
15	6.13	-0.73	177.1	-3.50	300.1934	0.1	$[M+Na]^+$	$C_{17}H_{27}NO_2$	2-Ethylhexyl 4-(dimethylamino)benzoate	30541	CPPdb/ FCCdb
16	6.25	-0.12	187.9	0.22	367.1875	-1.4	$[M+Na]^+$	$C_{21}H_{28}O_4$	Bisphenol A bis(2-hydroxypropyl) ether	8306	FCCdb
17	6.3	-0.38	189.1	-3.75	369.2239	-2.4	$[M+Na]^+$	$C_{18}H_{34}O_6$	Bis(2-butoxyethyl) adipate	8837	CPPdb
18	6.35	-1.11	224.2	2.86	509.3344	4.6	$[M+H]^+$	$C_{25}H_{48}O_{10}$	Pentanedioic acid, bis(2-(2-(2-butoxyethoxy)ethoxy)ethyl) ester	103407	CPPdb

19	6.40	-0.07	171.9	-0.08	327.0781	0.0	$[M+H]^+$	$C_{18}H_{15}O_4P$	Triphenyl phosphate	8289	CPPdb/FCCdb
20	6.62	-0.73	204.8	3.63	439.2672	1.2	$[M+Na]^+$	$C_{22}H_{40}O_7$	2-(2-(2-(Dodecyloxy)ethoxy)ethoxy)ethyl hydrogen maleate	6437561	FCCdb
21	6.72	0.46	181.5	-3.24	341.1278	-1.8	$[M+H]^+$	$C_{22}H_{16}N_2O_2$	3,10-Dimethylquinolino[2,3-B]Acridine-7,14(5H,12H)-Dione	11382230	CPPdb
22	6.87	-0.08	234.6	-2.31	553.3969	4.1	$[M+H]^+$	$C_{28}H_{56}O_{10}$	Octaethylene Glycol Laurate	10187601	FCCdb
23	6.87	0.01	245.4	-4.10	531.4149	3.2	$[M+Na]^+$	$C_{30}H_{56}N_2O_4$	Bis(1,2,2,6,6-Pentamethyl-4-Piperidyl) Sebacate	586744	CPPdb/FCCdb
24	7.27	1.27	202.9	2.03	465.2582	-1.2	$[M+Na]^+$	$C_{20}H_{43}O_8P$	3,6,9,12-Tetraoxatetracosan-1-ol, dihydrogen phosphate	170762	CPPdb/FCCdb
25	7.34	-0.27	194.3	-3.89	337.2134	-1.2	$[M+Na]^+$	$C_{21}H_{30}O_2$	Methyl dehydroabietate	14697	CPPdb
26	7.46	-0.51	204.3	2.93	375.2504	-0.4	$[M+Na]^+$	$C_{21}H_{36}O_4$	5-Carboxy-4-hexyl-2-cyclohexene-1-octanoic acid	105841	FCCdb
27	7.46	0.53	170.6	2.91	259.1691	-0.5	$[M+H]^+$	$C_{17}H_{22}O_2$	Geranyl benzoate	5353011	FCCdb
28	7.59	0.86	250.7	3.49	605.4233		$[M+Na]^+$	$C_{30}H_{62}O_{10}$	Polidocanol	656641	CPPdb/FCCdb
29	7.66	-0.39	196.8	0.74	349.2729	4.6	$[M+Na]^+$	$C_{20}H_{38}O_3$	Glycol oleate	5364420	CPPdb/FCCdb
30	7.82	-0.15	216.3	2.97	395.277	0.5	$[M+Na]^+$	$C_{21}H_{40}O_5$	Decanoic acid, ester with 1,2,3-propanetriol octanoate	19026760	FCCdb
31	7.83	0.78	176.0	2.53	273.1845	-1.3	$[M+H]^+$	$C_{18}H_{24}O_2$	Geranyl Phenylacetate	5366044	FCCdb
32	7.86	0.31	198.0	1.85	353.2659	-1.0	$[M+Na]^+$	$C_{19}H_{38}O_4$	Methyl 9,10-dihydroxyoctadecanoate	66194	CPPdb/FCCdb
33	8.16	0.39	212.2	3.44	397.3285	-0.9	$[M+Na]^+$	$C_{22}H_{46}O_4$	ceteth-3	4639427	FCCdb
34	8.2	1.02	229.3	-0.20	475.2374	-1.3	$[M+H]^+$	$C_{32}H_{30}N_2O_2$	9,10-Anthracenedione, 1,4-bis[(2-ethyl-6-methylphenyl)amino]-	6451771	FCCdb
35	8.24	0.06	203.4	0.77	312.3259	-0.6	$[M+Na]^+$	$C_{20}H_{41}NO$	icosanamide	3016647	CPPdb
36	8.26	-0.35	314.2	-0.83	844.6006	-3.7	$[M+Na]^+$	$C_{44}H_{88}NO_{10}P$	Hydroxylated lecithin	57508518	FCCdb
37	8.32	-0.21	237.0	-0.14	493.3503	0.7	$[M+Na]^+$	$C_{27}H_{50}O_6$	Glyceryl trioctanoate	10850	FCCdb
38	8.61	0.64	211.5	3.13	381.3339	-0.1	$[M+Na]^+$	$C_{22}H_{46}O_3$	Arosurf	10991978	FCCdb
39	8.74	1.05	203.0	1.72	351.2557	-1.9	$[M+H]^+$	$C_{18}H_{38}O_4S$	2-Hydroxy-1-Octadecanesulfonic Acid	414070	FCCdb

40	9.02	-0.73	277.0	0.00	619.527	-0.3	$[M+Na]^+$	$C_{37}H_{72}O_5$	Glycerides, C16-18 Mono- And Di-	3086206	CPPdb/FCCdb
41	9.02	-0.74	249.9	-1.01	533.4541	0.1	$[M+Na]^+$	$C_{32}H_{62}O_4$	Ditridecyl adipate	85653	CPPdb
42	9.21	-0.25	249.8	-1.41	547.3992	-0.2	$[M+H]^+$	$C_{33}H_{54}O_6$	Tris(2-ethylhexyl) trimellitate	18725	CPPdb/FCCdb
43	10.11	0.04	264.5	0.60	559.5177	0.7	$[M+Na]^+$	$C_{34}H_{68}N_2O_2$	Hexadecanamide, N,N'-1,2-ethanediylbis-	79654	CPPdb/FCCdb
44	11	0.83	272.1	-0.91	587.5487	0.2	$[M+Na]^+$	$C_{36}H_{72}N_2O_2$	Ethylene-N-palmitamide-N'-stearamide	101432497	CPPdb/FCCdb
